# Disrupted functional connectivity between perirhinal and parahippocampal cortices with hippocampal subfields in patients with mild cognitive impairment and Alzheimer's disease

**DOI:** 10.18632/oncotarget.17944

**Published:** 2017-05-16

**Authors:** Yu Sun, Yafei Wang, Jiaming Lu, Rengyuan Liu, Christopher G. Schwarz, Hui Zhao, Yue Zhang, Lingyi Xu, Bin Zhu, Bing Zhang, Bing Liu, Suiren Wan, Yun Xu

**Affiliations:** ^1^ School of Biological Sciences and Medical Engineering, Southeast University, Nanjing, China; ^2^ The Institute of Cancer and Genomics Sciences, University of Birmingham, Birmingham, U.K; ^3^ Department of Radiology, The Affiliated Drum Tower Hospital of Nanjing University Medical School, Nanjing, China; ^4^ Department of Neurology, The Affiliated Drum Tower Hospital of Nanjing University Medical School, Nanjing, China; ^5^ Department of Radiology, Mayo Clinic and Foundation, Rochester, MN, USA; ^6^ Brainnetome Center, Institute of Automation, Chinese Academy of Science, Beijing, China

**Keywords:** functional connectivity, perirhinal cortex, parahippocampal cortex, AD, MCI

## Abstract

Most patients with mild cognitive impairment and Alzheimer's disease can initially present memory loss. The medial temporal lobes are the brain regions most associated with declarative memory function. As sub-components of the MTL, the perirhinal cortex, parahippocampal cortex and hippocampus have also been identified as playing important roles in memory. The functional connectivity between hippocampus subfields and perirhnial cortices as well as parahippocampal cortices among normal cognition controls (NC group, n=33), mild cognitive impairment (MCI group, n=31) and Alzheimer's disease (AD group, n=27) was investigated in this study. The result shows significant differences of functional connectivity in 3 pairs of regions among NC group, MCI group and AD group: right perirhinal cortex with right hippocampus tail, left perirhinal cortex with right hippocampus tail, and right parahippocampal cortex with right hippocampus head. Clustering methods were used to classify NC group, MCI group and AD group (accuracy=100%) as well as different subtypes of mild cognitive impairment patients based on functional alterations. Functional connectivity disrupted between perirhinal and parahippocampal cortex with hippocampal subfields, which may provide a better understanding of the neurodegenerative progress of MCI and AD.

## INTRODUCTION

Alzheimer's disease (AD) is a common neurodegenerative disorder in the elderly. It accounts for 60% to 70% of dementia cases [1]. Mild cognitive impairment (MCI) has been seen as a prodromal stage of AD [1]. Studies suggest that patients with MCI tend to progress to probable AD at a rate of approximately 10-15% each year [2]. MCI can present a variety of symptoms, and the principal cognitive impairments include amnestic MCI (aMCI), single non-memory domain or multiple cognitive domains MCI, etc.[3, 4]. The subtype of aMCI will progress to AD with a relatively higher risk of 80% within 5 years [5]. Although aMCI patients may not meet neuropathologic criteria for AD, patients may be in a transitional stage of evolving AD. Patients in this hypothesized transitional stage demonstrated diffuse amyloid in the neocortex and frequent neurofibrillary tangles in the medial temporal lobe(MTL). Identifying sensitive markers in MCI populations can help detect early structural or functional alterations in the brain, which often exist before neuropathological damage when individuals are still functioning normally in their daily lives. It is crucial for early detection of disease and, ultimately, early intervention with disease-modifying therapy to slow or prevent cognition decline and thereby preserve quality of life [6].

Amyloid deposition and neurofibrillary tangles in the MTL are the most common pathological features of AD, which occur prior to memory loss symptoms [7]. The MTL are the brain regions most associated with declarative memory [8], including the hippocampus, entorhinal cortex, perirhinal cortex (PRC), and parahippocampal cortex (PHC) [8]. Previous studies have suggested that the PHC (located in the posterior parahippocampal gyrus) supports recollection by encoding and retrieving contextual information, whereas the hippocampus supports recollection by associating item and context information. By contrast, the PRC (located in the anterior parahippocampal gyrus and rhinal sulcus) supports familiarity by encoding and retrieving specific item information [9, 10]. The widely researched ectorhinal area is exactly lotated in the PRC. The pathway between the hippocampus and neocortical regions includes the entorhinal cortex and other MTL structures like PRC and PHC. Because these areas are first affected in AD, this disease has been thought to involve a breakdown in functional connectivity between the hippocampus and the rest regions of the brain [11]. Previously, Libby et al [12]had designed an experiment to study the functional connectivity between PHC and PRC with hippocampal subfields in 15 cognitively normal people. Libby et al [12] had two important findings. One is that PRC showed preferential connectivity with the anterior hippocampus, whereas PHC showed preferential connectivity with posterior hippocampus. The other is that these is significant preferential PRC connectivity with an anterior temporal and frontal cortical network, and preferential PHC connectivity with a posterior medial temporal, parietal, and occipital network in the 15 participants. However, the previous study only revealed the connectivity pattern in cognitively normal people. Little work has examined the possible functional connectivity alterations between the PRC and PHC with hippocampal subfields in patients with MCI and AD. The purpose of this study is to determine the different patterns of functional connectivity between PRC and PHC with three pairs of hippocampal subfields (head, body and tail from bilateral hippocampi) in cognitively normal control (NC), MCI and AD patients by using resting-state blood oxygen level–dependent (rs BOLD) MRI. We hypothesized that NC, MCI and AD patients would have different functional connectivity between these regions, and these differences might be a biomarker of AD. Moreover, these alterations would reveal gradually disrupted characteristics from NC to MCI and to AD patients. And then we expect to find a novel biomarker to distinguish MCI and AD patients and different subtypes of MCI.

## RESULTS

Demographic and clinical characteristics of 90 subjects are provided in Table 1. No significant difference was found among the three groups in age, gender or education background. The pathological alteration among groups led to the significant difference in the scores of MMSE and MOCA.

**Table 1:** The characteristics of patients are presented

	AD(n=27)	MCI(n=31)	NC(n=32)	p
**Age (years)**	74.04±11.57	73.39±10.39	68.66±11.78	0.130
**Gender(M/F)**	15/12	17/14	21/11	0.635
**Education(years)**	11.6±3.24	12.39±3.43	13.93±3.25	0.237
**MMSE**	17.43±5.830	25.75±2.303^a^	28.97±1.245^a,b^	<0.05^*^
**MOCA**	12.33±4.431	21.50±2.560^a^	27.04±1.990^a,b^	<0.05^*^

^*^: Significantly different among AD, MCI and NC at p < 0.05. Values are mean ±SD. MMSE: Mini-Mental State Examination.

*MOCA: Montreal Cognitive Assessment.^a^ Significant compared to NC. ^b^Significant compared to MCI*.

Functional connectivity from three pairs of regions (right PRC with right hippocampal tail, p=0.018; left PRC with right hippocampal tail, p=0.020; right PHC with right hippocampal head, p=0.004) were found to have a statistically significant difference in among the three groups of patients. Between right hippocampal tail with right and left PRC, there is a significant decline among three groups: the AD group have the lowest functional connectivity while the NC group have the highest value. However, between right hippocampal head right PHC, the AD group have a significant decrease in functional connectivity compared with NC (p=0.03) and MCI (p=0.003), while the MCI group has a slight increase compared with NC. The classification accuracy of NC, MCI and AD patients by the hierarchical clustering method reached 100%. GMM clustering method has found two Gaussian probability-density function curves, which revealed that two MCI subtypes were clearly distinguished by GMM.

## DISCUSSION

The purpose of this study was to find some sensitive markers to help the understanding of the neurodegenerative progress of MCI and AD. In our study, fMRI functional connectivity from three pairs of regions were found to have statistically significant differences among groups, including the right PRC with right hippocampal tail, left PRC with right hippocampal tail, and right PHC with right hippocampal head. The functional connectivities between these three pairs of regions were further used as discriminant features to classify NC, MCI and AD groups with an accuracy of 100% based on the Hierarchical clustering analysis method. K-means and GMM clustering also showed apparent distribution of the three groups, which could convince the significance of the functional connectivity alteration we found. The functional connectivity alternations can also be used as biomarkers to distinguish MCI patients into two subtypes by an automatic GMM clustering algorithm.

Most researches are focus on the functional connectivity between the whole hippocampus with other cortex, little work has been done about the possible functional connectivity alterations between different hippocampus subfields inpatients with MCI or AD. Even though Libby et al [12] have explored the connective pattern among hippocampus subfields in head, body and tail, it was only estimated in normal cognition control group. In our study, a semi-supervised clustering method was implemented to segment hippocampus head, body and tail. The significant differences among cognitive normal controls (NC) and patients with MCI as well as AD with hippocampus subfields may help to find the precise lesion of MCI or AD patients.

### Functional connectivity between PRC and PHC with hippocampal subfields

Previous studies have suggested that the MTL plays a vital role in memory encoding [9, 10]. The MTL structures, which have widespread and mutual connections with neocortex, are essential for building long-term memory for events and facts [8]. The PRC and PHC are two important components of the MTL. Scientists have found that the hippocampus and PHC are important for recollection but not familiarity, possibly via the representation and retrieval of contextual (especially spatial) information. On the contrary, the PRC contributes to and is necessary for familiarity-based recognition [9, 13]. Moreover, researchers found that, after the MTL is disrupted, recent memories are damaged but very remote memories are intact [23]. This corresponds with the features of memory impairment in AD and MCI patients that have short-term memory loss [24–26] at the initial stage, which suggests that the abnormal functional connectivity within the MTL may be related to AD and MCI. Our results reveal significant decreases in connectivity between the right PRC and right hippocampus tail and left PRC and right hippocampus tail in NC, MCI and AD groups (p=0.008, p=0.03, respectively, FDR corrected). This result demonstrates that with the disease progression, connectivity further decreases between the PRC and specific hippocampus sub-regions, affecting familiarity-based recognition. Between the right PHC and right hippocampus head sub-region, the MCI group have a slight increase compared with NC. In contrast, the AD group have a significant decrease compared to the NC and MCI groups. It appears that, early in the course of MCI when memory deficits are less prominent, there may be hyper-activation of MTL circuits, possibly representing inefficient compensatory mechanism for memory encoding activity [27, 29]. Moreover, we found that the right PHC and right hippocampus head have a decreased functional connectivity (p=0.022, FDR corrected). However, no such result has been previously reported according to our knowledge. We can only speculate that the impaired functional connectivity between PHC and hippocampus may indicate that during the process of AD, both familiarity-based recognition and rebased memory are impaired.

### Classification of different patient groups based on Functional connectivity

The alterations of functional connectivity within the MTL caused by pathological changes motivated us to try pattern recognition among different patient groups. Hierarchical clustering is a method of cluster analysis which seeks to build a hierarchy of clusters [27]. It is a “bottom up” approach where each subject starts in its own cluster, and pairs of clusters with the smallest distance are merged as one and then moves up the hierarchy tree. Hierarchical clustering has the distinct advantage that any valid measure of distance can be used. Ninety subjects were clearly distinguished with an accuracy of 100% among AD, MCI and NC groups.

### Classification of heterogeneity of MCI based on Functional connectivity

MCI is of strong interest to both clinicians and researchers, and is a concept encompassing much more than a preclinical state of AD [3]. When patients with MCI are followed over time, some develop AD or other dementia types, but some remain stable or even recover [28]. Moreover, MCI is a heterogeneous clinical entity with multiple etiologies [4] [28]. Based on diffusion tensor imaging (DTI), Kazuko et al [29] have divided MCI into two subtypes using fractional anisotropy (FA) and mean diffusivity (MD). Haobo Zhang et al [30] have discovered distinct grey matter (GM) atrophy patterns of different MCI subtypes based on voxel-wise GM volume from T1WI. Based on 18fluorodeoxyglucose (FDG)-positron emission tomography, researchers from Mayo Clinic have found different patterns of neurodegeneration caused by β-amyloidosis in MCI patients [31]. These findings provide evidence for the possibility to divide MCI into different subtypes which may improve the diagnosis of MCI. In our study, 31 MCI patients were recognized as two subtypes by GMM clustering according to the functional connectivity alterations within MTL. The result illustrated that these alterations may provide potential makers for early diagnosis of MCI.

## MATERIALS AND METHODS

### Subjects

This retrospective study was approved by the ethics committees of the Affiliated Drum Tower Hospital of Nanjing University Medical School. Between October 2010 and February 2014, a total of 90 subjects (age: 71.9±11.33 years, 27 AD, 31 MCI and 33 NC) were recruited from the Department of Neurology of the Affiliated Drum Tower Hospital of Nanjing University Medical School. All patients provided written informed consent and the study was conducted according to the provisions of the Helsinki declaration.

All subjects underwent a standardized process, including a neuropsychological screening, a whole brain MRI, a general medical, and a neurological examination that was performed by a neurologist. The clinical diagnosis of probable AD was confirmed by a multidisciplinary consensus meeting according to the National Institute of Neurological and Communicative Disorders and Stroke and the Alzheimer's Disease and Related Disorders Association (NINCDS-ADRDA) criteria [32]. It is worth noting that the AD and MCI groups have not been distinguished by any biomarker, such as PET or CSF beta-amyloid.

MCI was diagnosed according to the criteria [13, 14]. The criteria were as follows: cognitive concern reflecting a change in cognition reported by patient or informant or clinician; objective evidence of impairment in one or more cognitive domains, typically including memory; preservation of independence in functional abilities, and not demented. Individuals with any cerebrovascular abnormalities, as determined by T_2_WI, or a history of brain injury or alcoholism were excluded from the study. Subjects with visible white matter hyperintensity were also excluded. All of the normal control subjects had no cognitive complaints and no neurological or psychiatric disorders, with the same exclusion criteria as the AD and MIC patients. All participants were dextromanual.

### Neuropsychological testing

Cognitive testing was performed before the MRI examination. General cognitive status was evaluated by the Mini-Mental State Examination (MMSE) [15] and the Montreal Cognitive Assessment (MoCA) [16].

### Resting-state BOLD data acquisition

Data were acquired using a 3.0T MR system (Achieva 3.0T TX dual-source parallel RF excitation and transmission technology, Philips Medical Systems, The Netherlands) using an eight-channel phased array coil. We used an eight-channel phased-array head coil with foam padding and headphones to restrict head motion and scanner noise. Three-dimensional high-resolution sagittal T1W with turbo fast echo (3D-T_1_TFE) was performed (repetition time=9.7ms; echo time=4.6ms; in plane spatial resolution=1^*^1mm^2^; acquisition matrix=256^*^256; Flip Angel=8; slice thickness=1mm; acquisition time=5min). Resting-state BOLD was performed (repetition time=2000ms; echo time=30ms; in plane spatial resolution=3^*^3mm^2^; slice thickness=4mm; number of slices=35; time points=230; acquisition matrix=64^*^64; Flip Angel=90; slice thickness=4mm, acquisition time=8min). Participants were asked to lie still with their eyes closed but remaining awake. All images were visually inspected to ensure that no significant MRI artifacts exist. The head motions of all subjects were within 3mm and 3 degrees.

### Resting-state BOLD data processing

#### Pre-processing

The pre-processing work was running on the Brainnetome fMRI Toolkit (BRANT 2.0, http://www.brainnetome.org/en/brainnetometool/fmri-toolkit.html), The pre-processing pipeline is as followed: slice timing correction, realign for head movement, normalization into standard space, removal of physiological drifts and noise, band-pass filtering (0.01-0.08Hz) and smooth (Gaussian kernel with FWHM=4mm).

#### Masks of ROIs

The left and right PHC masks (PHC_L and PHC_R) are directly taken from AAL (Anatomical Automatic Labeling) Template (NO.39 and NO.40) [17]. The bilateral PRC (in the rhinal sulcus) masks are directly taken from Brodmann Template (NO.35 and NO.36) as a whole [18]. NO.36 Brodmann Template is widely known as the ectorhinal cortex (EC), which is now of part of the perirhinal cortex. The PRC masks were then segmented into left and right parts (PRC_L and PRC_R) manually on fMRI data processing software REST. The voxel resolution of PHC_L, PHC_R, PRC_L and PRC_R is completely the same: 2^*^2^*^2mm3. We segmented the hippocampus into head, body and tail with the help of Junjie Zhuo by using the method by Cheng et al [19]. They have proposed a semi-supervised clustering method for hippocampus segmentation. Consider each voxel as one node of the graph and then connect each pair of voxels with an edge weighted by a similarity measure between their functional signals. Using this, the hippocampus was segmented into functionally homogeneous subfields (head, body and tail) based on resting state fMRI data of 28 healthy subjects. The resulting hippocampal geometric parcellation is adopted as prior information, and a spatially consistent constraint is adopted as a regularization term to achieve spatially contiguous clustering. All hippocampal subfields were then resliced according to PHC and PRC masks: voxel resolution=2^*^2^*^2mm^3^.

As the masks we collected were extracted from different templates or by different methods, there might be scaling differences in masks. To minimize the influence of scaling differences and to minimize the individual variations among different people, we carried out a spherical process on each mask (running on REST software). Firstly, we manually selected the ROI center of each mask according to the sagittal, coronal and cross section images. This ROI center was considered as the center of the ball. Then, the radius of the ball was set respectively. ALL masks are re-designed into a smaller ball with radius of 3mm, except PHC_L and PHC_R, the radius of which was 4mm. At last, the re-designed sphere masks were used for functional connectivity analyses.

#### Functional connectivity

Functional Connectivity was also calculated using the BRAT 1.0 software with the pre-processed data. Based on the different masks we obtained, we examined four groups of functional connectivity combinations: 1). PRC_R with bilateral hippocampal subfields (Head_R, Body_L, Tail_R, Head_L, Body_L, Tail_L); 2). PRC_L with bilateral hippocampal subfields; 3). PHC_R with bilateral hippocampal subfields; 4). PHC_L with bilateral hippocampal subfields.

### Statistical analysis

Statistical analysis was performed using SPSS (V 21.0), including a One-way analysis of variance (ANOVA) among NC, MCI and AD groups with *p* < 0.05, and a One Sample *t*-test between the two patient groups with *p* < 0.05. Multiple comparisons correction was performed using MATLAB code based on the FDR (False discovery rate) principle.

### Clustering analysis

Three kinds of clustering methods were used to cluster AD, MCI and NC groups, hierarchical clustering analysis, Gaussian mixture method and K-means clustering. These three methods were then used to recognize different MCI subtypes. Clustering results with the best accuracy was considered as the optimal method.

In hierarchical clustering analysis (HCA), the functional connectivity between the PRC_R and Tail_R), PRC_L and Tail_R, and PHC_R and Head_R were taken as coordination values, and each subject can be seen as a point (P) located in a multidimensional space, P(x,y,z) = (PRC_R ~ Tail_R, PRC_L ~ Tail_R, PHC_R ~ Head_R). The similarity between subject *i* and subject *j* can be defined by the Euclidean Distance:

The smaller the distance is, the more similar two subjects are. The most similar subjects are identified as one cluster and the distance between two clusters is the minimal distance within two subjects from two clusters. This method is also called single-linkage HCA [20]. The similarity between two subjects is then used for HCA to cluster the AD, MCI and NC groups.

Gaussian mixture method (GMM) is based on the Gaussian probability density distribution to recognize these two different MCI subtypes. GMM is used to make statistical inferences about the properties of the sub-populations given only observations on the pooled population, without sub-population identity information. K-means clustering aims to partition all observations into k clusters in which each observation belongs to the cluster with the nearest mean, serving as a prototype of the cluster.

### Masks of hippocampal subfields

**Figure 1:**
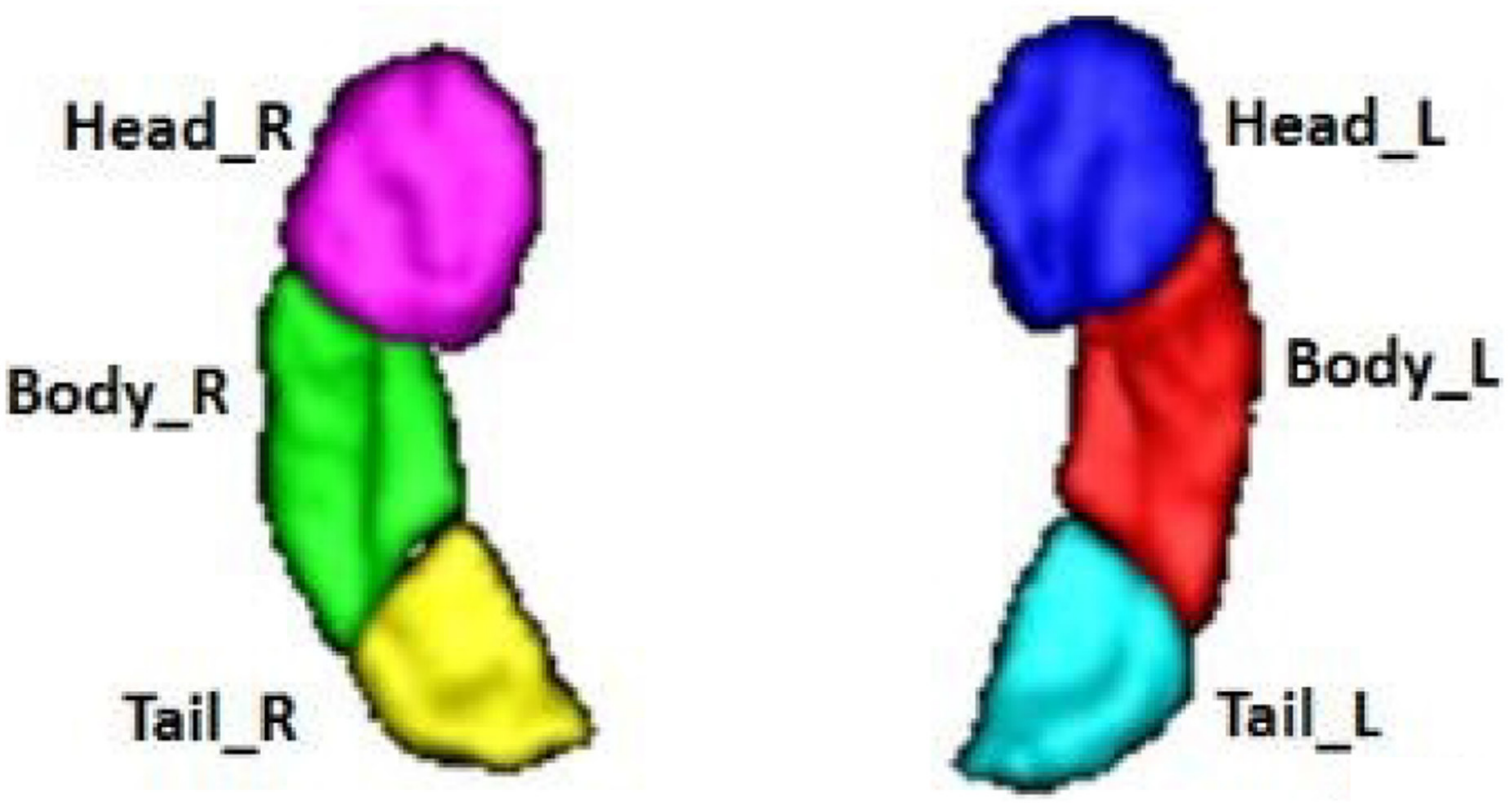
Segmentation of the bilateral hippocampi, which shows the volume rendering of the parcellation results Subfields are denoted by different colors. Head_R: right hippocampus head. Head_L: left hippocampus head. Body_R: right hippocampus body. Body_L: left hippocampus body. Tail_R: right hippocampus tail. Tail_L: left hippocampus tail.

**Figure 2:**
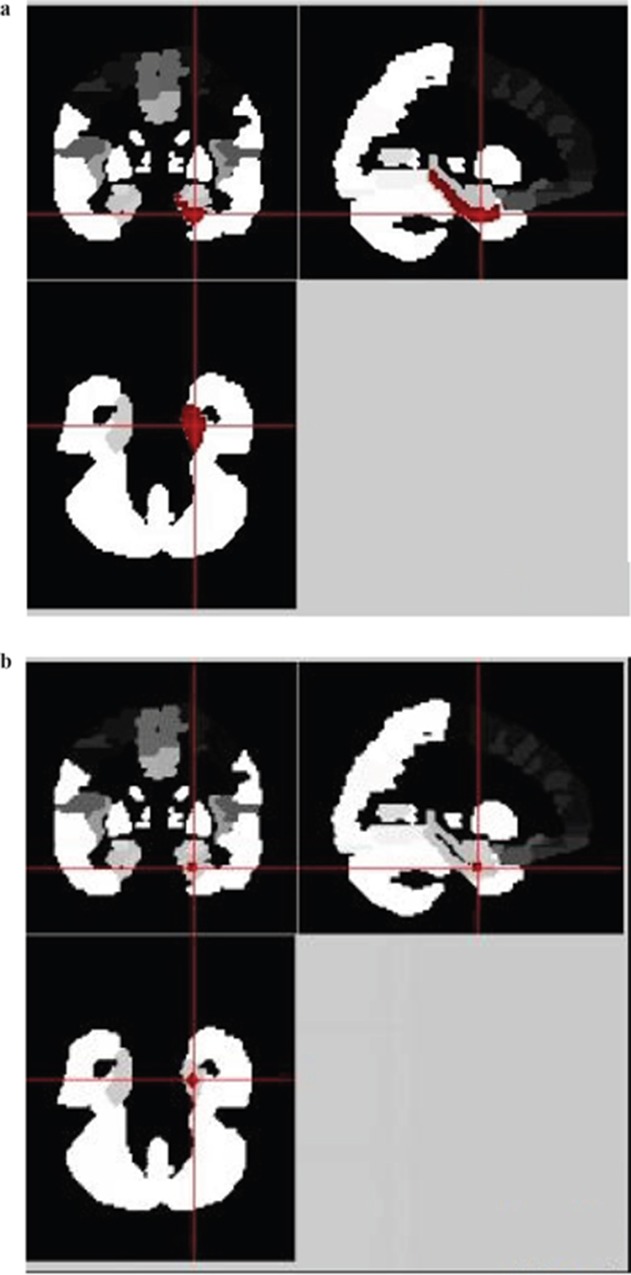
The spherical process of the left parahippocampal cortex (PHC_L) in REST **(a)** The irregular regions in red is the original PHC_L mask which is shown in sagittal, coronal and cross section. **(b)** The sphered regions in red is the re-designed Head_R mask shown in sagittal, coronal and cross section.

Using the semi-supervised clustering [19], we found 3 pairs of hippocampal subfields: left hippocampus head (Head_L), right hippocampus head (Head_R), left hippocampus body (Body_L), right hippocampus body (Body_R), left hippocampus tail (Tail_L) and hippocampus tail_R (Tail_R). The segmentation results, which are shown in Figure 1, were confirmed by an experienced neuroradiologist. The central coordination of each ROI are given in Table 2. Each hippocampal subfield, as well as the bilateral PRC and PHC ROIs, was re-designed into a ball by REST. For instance, the sphereical mask of the left parahippocampal cortex (PHC_L) is shown as Figure 2.

**Table 2:** The central coordination information of each ROI

	X(mm)	Y(mm)	Z(mm)
**PRC_R**	20	-1	-33
**PRC_L**	-20	-1	-33
**PHC_R**	23	-5	-29
**PHC_L**	-23	-5	-29
**Head_R**	27	-15	-21
**Head_L**	-27	-15	-21
**Body_R**	29	-27	-12
**Body_L**	-29	-27	-12
**Tail_R**	27	-35	-5
**Tail_L**	-27	-35	-5

### Functional connectivity

**Figure 3:**
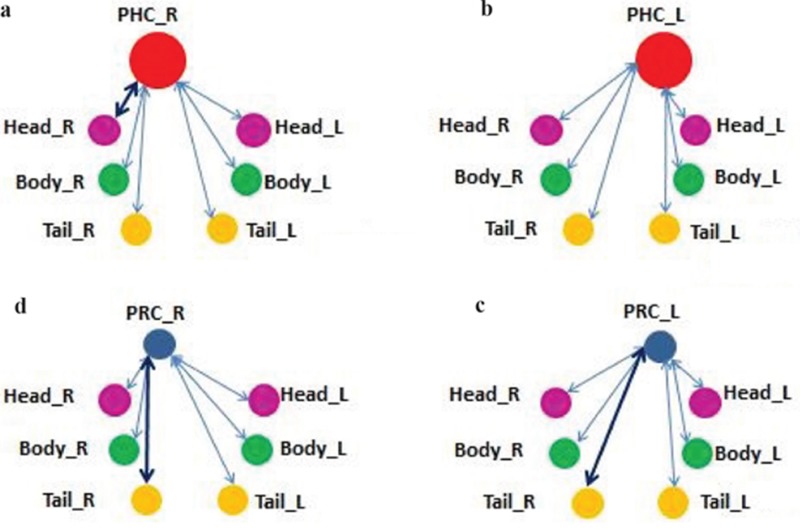
Four functional connectivity combinations Figure **(a)** and **(b)** are the connecting between PRC ROIs with 6 hippocampal subfields. Figure **(c)** and **(d)** are connecting between PHC ROIs with 6 hippocampal subfields.

Four groups of functional connectivity combinations are shown in Figure 3. Each ROI displays as a ball after the spherical process. Combinations with significant statistics differences are connected with bold black lines while others are connected with blue lines. The statistical results are shown in Table 3. Three pairs of regions were found significantly different among NC, MCI and AD: right PRC with right hippocampus tail (PRC_R ~ Tail_R), left PRC with right hippocampus tail (PRC_L ~ Tail_R), and right PHC with right hippocampus head (PHC_R ~ Head_R). The statistical results of these three pairs of regions were then given a multiple comparisons correction based on FDR. The corrected *p* values after FDR are shown in column 7. Two pairs of regions were still found to have significant differences after FDR multiple comparisons correction: left PRC with right hippocampus tail (PRC_L ~ Tail_R), and right PHC with right hippocampus head (PHC_R ~ Head_R).

**Table 3:** The Functional Connectivity between different ROIs

Connecting Masks		NC	MCI	AD	ANOVA p value	FDR p value
PRC_R~	Head_R	0.067±0.22	0.122±0.18	0.101±0.23	0.582	--
	Body_R	0.033±0.23	0.060±0.23	0.127±0.21	0.263	--
	Tail_R	0.152±0.18	0.052±0.19	0.017±0.19	0.018^*^	0.008
	Head_L	0.081±0.24	0.050±0.25	0.087±0.20	0.807	--
	Body_L	0.106±0.21	0.050±0.18	0.038±0.22	0.374	--
	Tail_L	0.122±0.26	0.064±0.22	0.028±0.29	0.372	--
PRC_L~	Head_R	0.006±0.19	0.037±0.19	-0.003±0.21	0.718	--
	Body_R	0.048±0.24	0.068±0.21	0.093±0.20	0.716	--
	Tail_R	0.075±0.17	0.067±0.19	-0.049±0.18	0.020^**^	0.022
	Head_L	-0.022±0.18	0.008±0.20	-0.034±0.26	0.460	--
	Body_L	0.028±0.22	0.030±0.16	0.062±0.20	0.357	--
	Tail_L	-0.014±0.26	0.773±0.17	0.044±0.19	0.322	--
PHC_R~	Head_R	0.057±0.17	0.118±0.20	-0.621±0.24	0.004^**^	0.03
	Body_R	0.087±0.22	0.210±0.30	0.173±0.21	0.137	--
	Tail_R	0.099±0.19	0.103±0.20	0.025±0.30	0.364	--
	Head_L	0.032±0.21	0.033±0.23	-0.05±0.25	0.784	--
	Body_L	0.182±0.18	0.198±0.25	0.125±0.23	0.336	--
	Tail_R	0.023±0.22	0.121±0.23	0.117±0.37	0.294	--
PHC_L~	Head_R	0.048±0.19	0.078±0.21	0.053±0.22	0.833	--
	Body_R	0.089±0.20	0.150±0.18	0.075±0.30	0.369	--
	Tail_R	0.086±0.16	0.060±0.21	0.084±0.24	0.426	--
	Head_L	0.030±0.19	-0.003±0.20	0.041±0.21	0.653	--
	Body_L	0.105±0.17	0.094±0.21	0.094±0.20	0.299	--
	Tail_R	0.091±0.20	0.091±0.14	0.025±0.29	0.411	--

*Values are mean ±SD*.

^*^: Significantly different among AD, MCI and NC at p < 0.05.

^**^: Significantly different among AD, MCI and NC after FDR correction at ANOVA p value< FDR p value.

**Figure 4:**
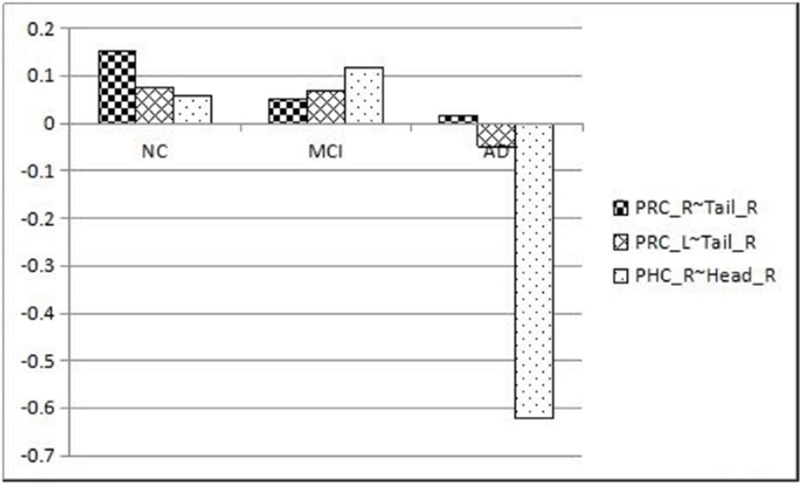
Different functional connectivity among the NC, MCI and AD groups

The mean functional connectivity values in the three pairs of masks (AVOVA *p* < 0.05) among NC, MCI and AD are demonstrated by the line chart in Figure 4. Between PRC_R and Tail_R, PRC_L and Tail_R, there is a significant decline among three groups: the AD group have the lowest functional connectivity while the NC group have the highest value. However, between the PHC_R and Head_R, the AD group have a significant decrease in functional connectivity compared with NC (p=0.03) and MCI (p=0.003), while the MCI group has a slight increase compared with NC.

As for these three pairs of ROIs with significant differences, we also had a One Sample t-test between each of the two patient groups. No statistically significant difference of functional connectivity was found between the PRC_R and Tail_R in AD and MCI patients, and no statistically significant difference was found between the PHC_R and Head_R in NC and MCI patients. Except for the above two pairs of masks, statistically significant differences with *p*<0.05 were found between each of the two groups as is shown in Table 4.

**Table 4:** One sample t-test of function connectivity between each two patient groups

Connecting Masks	t-test p(AD-MCI)	t-test p(AD-NC)	t-test p(MCI-NC)
PRC_R ~ Tail_R	0.484	0.008^*^	0.036^*^
PRC_L ~ Tail_R	0.022^*^	0.010^*^	0.022^*^
PHC_R ~ Head_R	0.003^*^	0.03^*^	0.193

Note: The symbol “~“denotes function connectivity between each pair. The symbol “^*^“ indicates p values <0.05.

### Clustering analysis in among AD, MCI and NC groups

**Figure 5:**
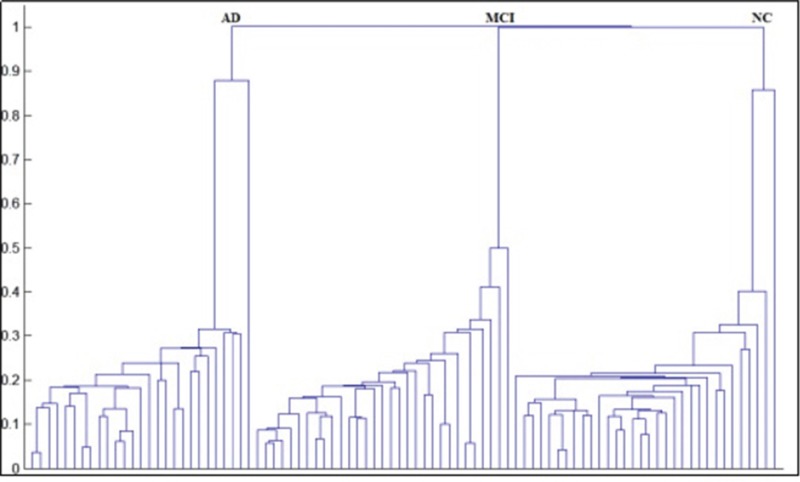
Hierarchical clustering chart of among AD, MCI and NC groups

The distance between two subjects was used to establish the hierarchical clustering tree, which is shown in Figure 5. The vertical axis is the distance between two subjects or clusters, and the horizontal axis represents the index of each subject (in our study, there are 90 subjects in total and therefore there are 90 points on the horizontal axis). The AD, MCI and NC groups are 100% correctly separated into their own clusters.

The HCA result has confirmed the clinical significance of the functional connectivity between the PRC_R and Tail_R, PRC_L and Tail_R, and PHC_R and Head_R. The alterations in these three pairs of regions can be considered as pathological changes and could potentially help with the diagnosis of Alzheimer's disease.

**Figure 6:**
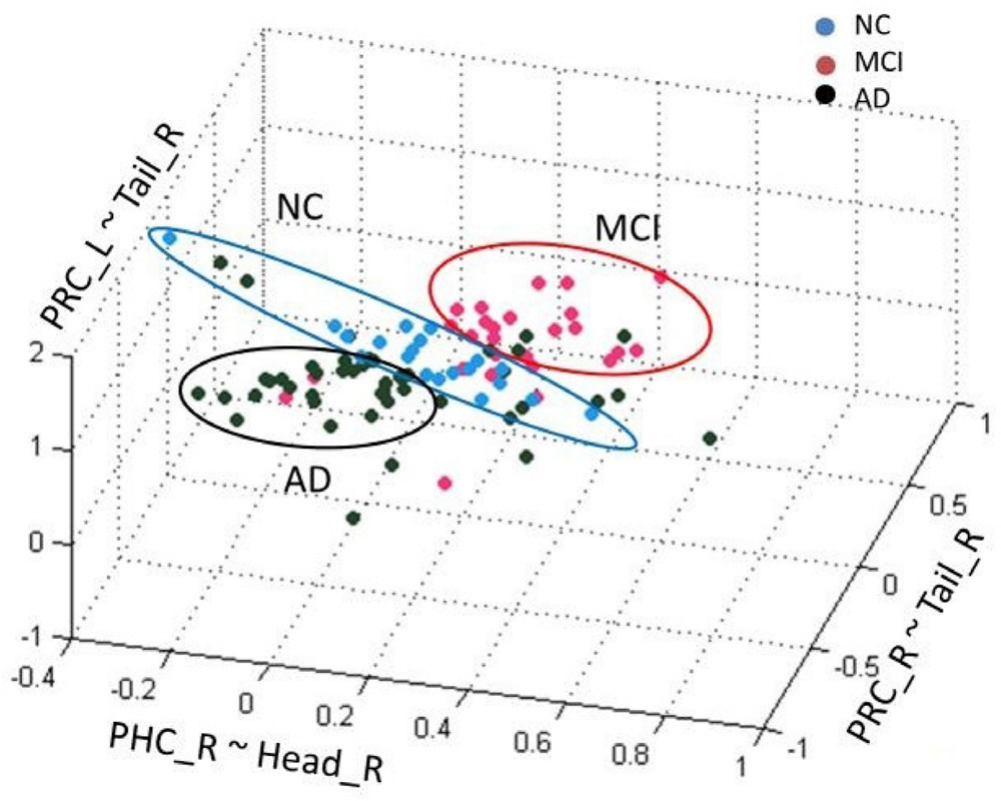
Gaussian mixture method of among AD, MCI and NC groups

**Figure 7:**
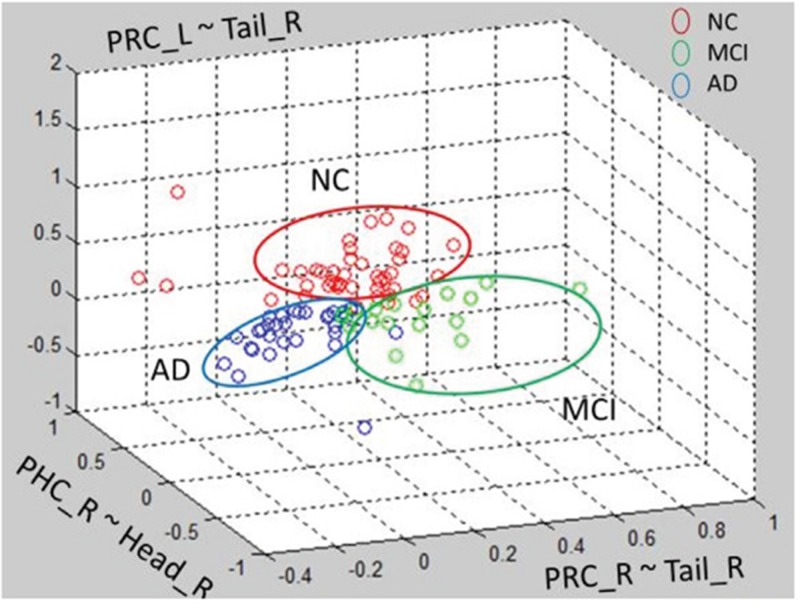
K-means clustering method of among AD, MCI and NC groups

Besides HCA, we also tied Gaussian mixture method (GMM) clustering and K-means clustering to recognize NC, MCI and AD patients (Figure 6 and Figure 7). Subjects were clearly divided into three clusters

Although GMM and K-means clustering can clearly divide subjects into three groups, the clustering accuracy is not as high as HCA. GMM had an accuracy of 42% and K-means 40%. Therefore, HCA could be the potential method for NC, MCI and AD classification in clinic.

### Gaussian mixture model clustering in different MCI subtypes

In 2001, Petersen, et al. have divided MCI into different subtypes including amnestic (aMCI), non-memory (nmMCI), and multi-domain (mMCI) [21]. And in 2006, Portet, et al. divided MCI into aMCI and nmMCI: two groups, where each group contains single-domain and multi-domain [22].

**Figure 8:**
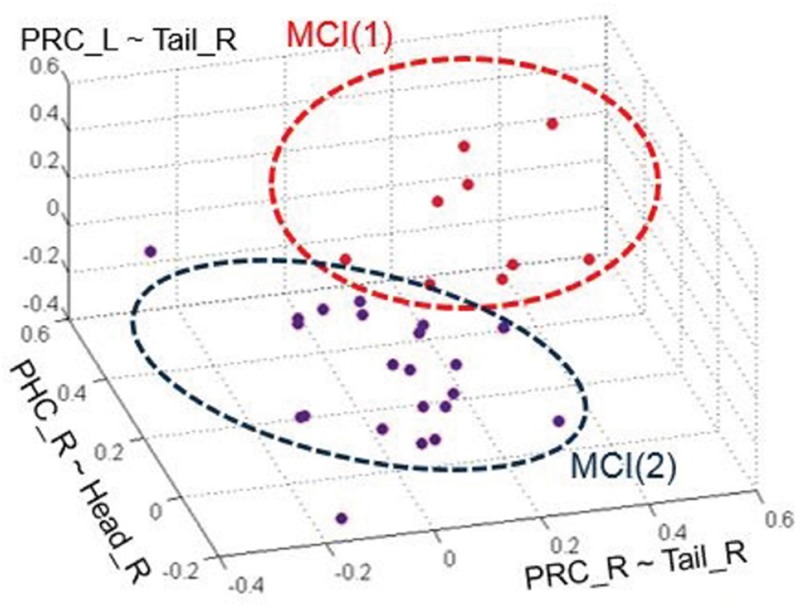
The clustering chart of two groups of MCI by GMM The red and blue groups represent each of the MCI subtypes, respectively.

Our HCA result among NC, MCI and AD patients suggested that we can use a clustering algorithm to recognize different MCI subtypes automatically according to connection patterns in the MCI group. We performed a pilot trial using GMM recognize MCI subtypes since it got better clustering results than HCA and K-means in MCI groups which have the most similar characteristics. As is shown is Figure 8, 31 MCI patients were clearly distinguished into two groups, MCI(1) and MCI(2), when using the functional connectivity between PRC_R and Tail_R, PRC_L and Tail_R, and PHC_R and Head_R as discriminant features. MCI(1) and MCI(2) are two clusters that have two different Gaussian probability-density function curves, and the different Gaussian probability density represent different subtypes' distribution. However, due to lack of sufficient historical clinical information, the diagnosed type of each MCI patient was unknown, and thus we could not determine the accuracy of the clustering. However, this suggests another potential objective measurement criterion for MCI classification by fMRI.

### Limitations and future directions

Our results indicate significant differences of functional connectivity between bilateral PRC and right hippocampus tail, right PHC and right hippocampus head among AD, MCI and NC subjects. The result may reveal some neuronal circuit alterations with the disease evolvement. These alterations could be used as discriminant features to classifiy AD, MCI and NC groups as well as different subtypes of MCI patients.

There are several limitations in the study. Firstly, hippocampal subfield masks were semi-supervised clustered from 28 healthy people. This method has been validated by comparing the functional and structural connectivity patterns of these subfields. These three pairs of hippocampal subfields have distinctive structural connectivity patterns, indicating that the hippocampal subfields identified using this method were structurally meaningful too. However, a bigger dataset with multi-modality MR information would be necessary to optimize the cluttering method. Secondly, different methods of masks segmentation might have led to scaling differences or individual variations which can influence functional connectivity analysis. We have re-designed each mask into a small sphere to help reduce the scaling difference or individual variation. Nevertheless, hippocampal subfields tend to be smaller than normal fMRI masks, and our smoothing with a 6mm FWHM of Gaussian kernel, which is commonly used in fMRI data, might be relatively large and might eliminate the heterogeneity of small masks. Further research is needed to find the optimal radius and smoothing window of each mask. Thirdly, in our study, the AD and MCI groups were diagnosed by a standardized process without having metabolic biomarker assessment like PET or CSF beta-amyloid and thus some patients might be divided into the wrong group. Future study should refer more clinical parameters to improve the reliability of the original dataset. Moreover, the clinically diagnosed subtypes of each MCI patient remained unknown, and thus follow-up visits are needed to determine the accuracy for classifying MCI subtypes. A more integral experiment with complete clinical information is needed to verify the usefulness of this GMM method, and to confirm the significance of functional connectivity alterations of the three pairs of regions in MCI subtypes research. Finally, the classification accuracy is cross - sectional data only. Although the result can help understand the neurodegenerative progress of AD, a bigger dataset of participants is demanded to help the results more robust.

## Abbreviations

MCI: mild cognitive impairment
AD: Alzheimer's disease
MTL: medial temporal lobes
PRC: perirhinal cortex
PHC: parahippocampal cortex
BOLD: blood oxygen level–dependent
HCA: hierarchical clustering analysis
GMM: Gaussian mixture model
NC: normal control
MMSE: Mini-Mental State Examination
MoCA: Montreal Cognitive Assessments

## References

[1] Burns A, Iliffe S, Maurer K (2009). Alzheimer's disease. BMJ.

[2] Grundman M, Petersen RC, Ferris SH, Thomas RG, Aisen PS, Bennett DA, Foster NL, Jack CR, Galasko DR, Doody R, Kaye J, Sano M, Mohs R, Alzheimer's Disease Cooperative Study (2004). Mild cognitive impairment can be distinguished from Alzheimer disease and normal aging for clinical trials. Arch Neurol.

[3] Winblad B, Palmer K, Kivipelto M, Jelic V, Fratiglioni L, Wahlund LO, Nordberg A, Bäckman L, Albert M, Almkvist O, Arai H, Basun H, Blennow K (2004). Mild cognitive impairment—beyond controversies, towards a consensus: report of the International Working Group on Mild Cognitive Impairment. J Intern Med.

[4] Lopez OL (2013). Mild cognitive impairment. Continuum (Minneap Minn).

[5] Petersen RC, Doody R, Kurz A, Mohs RC, Morris JC, Rabins PV, Ritchie K, Rossor M, Thal L, Winblad B (2001). Current concepts in mild cognitive impairment. Arch Neurol.

[6] DeKosky ST, Marek K (2003). Looking backward to move forward: early detection of neurodegenerative disorders. Science.

[7] Tiraboschi P, Hansen LA, Thal LJ, Corey-Bloom J (2004). The importance of neuritic plaques and tangles to the development and evolution of AD. Neurology.

[8] Squire LR, Zola-Morgan S (1991). The medial temporal lobe memory system. Science.

[9] Eichenbaum H, Yonelinas AP, Ranganath C (2007). The medial temporal lobe and recognition memory. Annu Rev Neurosci.

[10] Diana RA, Yonelinas AP, Ranganath C (2007). Imaging recollection and familiarity in the medial temporal lobe: a three-component model. Trends Cogn Sci.

[11] Allen G, Barnard H, McColl R, Hester AL, Fields JA, Weiner MF, Ringe WK, Lipton AM, Brooker M, McDonald E, Rubin CD, Cullum CM (2007). Reduced hippocampal functional connectivity in Alzheimer disease. Arch Neurol.

[12] Libby LA, Ekstrom AD, Ragland JD, Ranganath C (2012). Differential connectivity of perirhinal and parahippocampal cortices within human hippocampal subregions revealed by high-resolution functional imaging. J Neurosci.

[13] Petersen RC (2004). Mild cognitive impairment as a diagnostic entity. J Intern Med.

[14] Albert MS, DeKosky ST, Dickson D, Dubois B, Feldman HH, Fox NC, Gamst A, Holtzman DM, Jagust WJ, Petersen RC, Snyder PJ, Carrillo MC, Thies B, Phelps CH (2011). The diagnosis of mild cognitive impairment due to Alzheimer's disease: recommendations from the National Institute on Aging-Alzheimer's Association workgroups on diagnostic guidelines for Alzheimer's disease. Alzheimers Dement.

[15] Tombaugh TN, McIntyre NJ (1992). The mini-mental state examination: a comprehensive review. J Am Geriatr Soc.

[16] Smith T, Gildeh N, Holmes C (2007). The Montreal Cognitive Assessment: validity and utility in a memory clinic setting. Can J Psychiatry.

[17] Tzourio-Mazoyer N, Landeau B, Papathanassiou D, Crivello F, Etard O, Delcroix N, Mazoyer B, Joliot M (2002). Automated anatomical labeling of activations in SPM using a macroscopic anatomical parcellation of the MNI MRI single-subject brain. Neuroimage.

[18] Brodmann K (1909). Beiträge zur histologischen Lokalisation der Grosshirnrinde: VI. Die Cortexgliederung des Menschen. J Psychol Neurol.

[19] Cheng HW, Fan Y (2014). Functional parcellation of the hippocampus by clustering resting state fMRI signals. International Symposium on Biomedical Imaging.

[20] Murtagh F, Contreras P (2012). Algorithms for hierarchical clustering: an overview. Wiley Interdiscip Rev Data Min Knowl Discov.

[21] Petersen RC, Stevens JC, Ganguli M, Tangalos EG, Cummings JL, DeKosky ST (2001). Practice parameter: early detection of dementia: mild cognitive impairment (an evidence-based review). Report of the Quality Standards Subcommittee of the American Academy of Neurology. Neurology.

[22] Portet F, Ousset PJ, Visser PJ, Frisoni GB, Nobili F, Scheltens P, Vellas B, Touchon J, MCI Working Group of the European Consortium on Alzheimer's Disease (EADC) (2006). Mild cognitive impairment (MCI) in medical practice: a critical review of the concept and new diagnostic procedure. Report of the MCI Working Group of the European Consortium on Alzheimer's Disease. J Neurol Neurosurg Psychiatry.

[23] Simons JS, Spiers HJ (2003). Prefrontal and medial temporal lobe interactions in long-term memory. Nat Rev Neurosci.

[24] Alvarez P, Squire LR (1994). Memory consolidation and the medial temporal lobe: a simple network model. Proc Natl Acad Sci USA.

[25] Bäckman L, Jones S, Berger AK, Laukka EJ, Small BJ (2004). Multiple cognitive deficits during the transition to Alzheimer's disease. J Intern Med.

[26] Arnáiz E, Almkvist O (2003). Neuropsychological features of mild cognitive impairment and preclinical Alzheimer's disease. Acta Neurol Scand Suppl.

[27] Rokach L, Maimon O (2005). Clustering methods. Data mining and knowledge discovery handbook.

[28] Terry DP, Sabatinelli D, Puente AN, Lazar NA, Miller LS (2015). A Meta-Analysis of fMRI Activation Differences during Episodic Memory in Alzheimer's Disease and Mild Cognitive Impairment. J Neuroimaging.

[29] Hiyoshi-Taniguchi K, Oishi N, Namiki C, Miyata J, Murai T, Cichocki A, Fukuyama H (2015). The Uncinate Fasciculus as a Predictor of Conversion from Amnestic Mild Cognitive Impairment to Alzheimer Disease. J Neuroimaging.

[30] Zhang H, Sachdev PS, Wen W, Kochan NA, Crawford JD, Brodaty H, Slavin MJ, Reppermund S, Draper B, Zhu W, Kang K, Trollor JN (2012). Gray matter atrophy patterns of mild cognitive impairment subtypes. J Neurol Sci.

[31] Knopman DS, Jack CR, Lundt ES, Wiste HJ, Weigand SD, Vemuri P, Lowe VJ, Kantarci K, Gunter JL, Senjem ML, Mielke MM, Machulda MM, Roberts RO (2015). Role of β-Amyloidosis and Neurodegeneration in Subsequent Imaging Changes in Mild Cognitive Impairment. JAMA Neurol.

[32] Surhone LM, Tennoe MT, Henssonow SF (2010). NINCDS-ADRDA Alzheimer's Criteria.

